# EVALUATING ENHANCEMENTS TO ADDRESS SAMPLE DIVERSITY, RESPONDENT BURDEN, AND DIFFICULTY REACHING RESPONDENTS

**DOI:** 10.1093/geroni/igz038.2162

**Published:** 2019-11-08

**Authors:** Melissa Hobbs, Melissa Hobbs, Angela Greene, Christine Caffrey, Manisha Sengupta, Lauren Harris-Kojetin

**Affiliations:** 1 RTI International, RTP, North Carolina, United States; 2 RTI International, Research Triangle Park, North Carolina, United States; 3 NCHS, Hyattsville, Maryland, United States

## Abstract

Every two years since 2012, the National Study of Long-Term Care Providers includes provider surveys with residential care communities (RCCs) and adult day services centers (ADSCs), via a multi-mode approach using Web, hard-copy questionnaires, and computer-assisted telephone interviewing. In each wave, we struggled to achieve target response rates. First, diversity among providers surveyed—e.g. RCC size, type of ADSC—presents unique challenges. For RCCs, small communities have lower response compared to larger ones. For ADSCs, how they define themselves (medically vs socially oriented) influenced their decision to participate. Second, respondents’ perceived burden, particularly the time required to complete the survey, is a recurring concern especially for directors of multiple RCCs and ADSCs. Finally, reaching target respondents—directors, administrators or operators—is problematic. These challenges affect data quality. In this presentation, we share results of our efforts to enhance contacting and interviewing protocols intended to address low participation associated with these challenges.

